# MACHINE LEARNING–ENHANCED FRAILTY ASSESSMENT FOR HIGH-RISK OLDER PATIENTS UNDERGOING TAVR

**DOI:** 10.1093/geroni/igae098.0439

**Published:** 2024-12-31

**Authors:** Mamoun Mardini, Chen Bai, Catherine Price, Todd Manini, Mohammad Al-Ani

**Affiliations:** University of Florida, Gainesville, Florida, United States; University of Florida, Gainesville, Florida, United States; University of Florida, Gainesville, Florida, United States; University of Florida College of Medicine, Gainesville, Florida, United States; University of Florida, Gainesville, Florida, United States

## Abstract

Frailty assessment in hospital settings is growing as a method for patient selection, estimating adverse outcomes, and care planning. However, due to time and resource constraints, current frailty assessments are unstandardized and challenging to apply in clinical settings. This study aims to test frailty assessments by combining real-world structured and unstructured data using machine learning (ML) in older patients undergoing Transcatheter Aortic Valve Replacement (TAVR). We extracted data from 131 TAVR patients collected between January 2018 to December 2019, employing the Fried criterion (weight loss, exhaustion, walking speed, grip strength, and physical activity). We used Latent Dirichlet Allocation (LDA) to analyze clinical notes collected 60 days before the TAVR procedure, generating 150 distinct topics. Additionally, we extracted preoperative data from structured EHRs, including patient details up to six months prior to their frailty evaluation. This includes sociodemographic data, medical history, condition severity, and the latest biochemical markers. We merged LDA-generated topics with EHR features, applied LASSO regression to select the most relevant features, and used XGBoost for predicting frailty levels in patients. We validated our models using nested cross-validation. Integrating clinical notes and structured EHR significantly enhanced model performance, with an area under the curve (AUC) of 0.82, surpassing the EHR-only model’s AUC of 0.64. SHAP analysis, a method to explain the output of ML, identified key frailty predictors such as congestive heart failure management, back problems, and Atrial Fibrillation. This approach demonstrates significant potential in standardizing frailty assessments using real-world data and optimizing TAVR patient selection

